# Treatment of secondary hyperparathyroidism in haemodialysis patients: a randomised clinical trial comparing paricalcitol and alfacalcidol

**DOI:** 10.1186/1471-2369-10-28

**Published:** 2009-09-24

**Authors:** Ditte Hansen, Lisbet Brandi, Knud Rasmussen

**Affiliations:** 1 Medical Department, Roskilde Hospital, Koegevej 7-13, DK-4000 Roskilde, Denmark; 2 Department of Surgery and Internal Medicine, University of Copenhagen, Blegdamsvej 3B, DK-2200 Copenhagen, Denmark

## Abstract

**Background:**

Secondary hyperparathyroidism is a common feature in patients with chronic kidney disease. Its serious clinical consequences include renal osteodystrophy, calcific uremic arteriolopathy, and vascular calcifications that increase morbidity and mortality.

Reduced synthesis of active vitamin D contributes to secondary hyperparathyroidism. Therefore, this condition is managed with activated vitamin D. However, hypercalcemia and hyperphosphatemia limit the use of activated vitamin D.

In Denmark alfacalcidol is the primary choice of vitamin D analog.

A new vitamin D analog, paricalcitol, may be less prone to induce hypercalcemia and hyperphosphatemia.

However, a randomised controlled clinical study comparing alfacalcidol and paricalcitol has never been performed.

The primary objective of this study is to compare alfacalcidol and paricalcitol. We evaluate the suppression of the secondary hyperparathyroidism and the tendency towards hyperphosphatemia and hypercalcemia.

**Methods/Design:**

This is an investigator-initiated cross-over study. Nine Danish haemodialysis units will recruit 117 patients with end stage renal failure on maintenance haemodialysis therapy.

Patients are randomised into two treatment arms. After a wash out period of 6 weeks they receive increasing doses of alfacalcidol or paricalcitol for a period of 16 weeks and after a further wash out period of 6 weeks they receive the contrary treatment (paricalcitol or alfacalcidol) for 16 weeks.

**Discussion:**

Hyperparathyroidism, hypercalcemia and hyperphosphatemia are associated with increased cardiovascular mortality in patients with chronic kidney disease.

If there is any difference in the ability of these two vitamin D analogs to decrease the secondary hyperparathyroidism without causing hypercalcemia and hyperphosphatemia, there may also be a difference in the risk of cardiovascular mortality depending on which vitamin D analog that are used. This has potential major importance for this group of patients.

**Trial registration:**

ClinicalTrials.gov NCT004695

## Background

Secondary hyperparathyroidism is a common complication of chronic kidney disease that is mainly caused by decreased synthesis of 1,25(OH)_2_vitamin D and phosphate retention due to reduced kidney mass, and the resultant hypocalcemia.

Treatment involves suppression of the parathyroid hormone (PTH) production by restoration of 1,25(OH)_2_vitamin D levels by replacement therapy [1]. However, 1,25(OH)_2_vitamin D stimulates intestinal phosphate and calcium absorption as well as phosphate and calcium mobilisation from bone, leading to an increased risk of hyperphosphatemia and hypercalcemia. Hyperphosphatemia and hypercalcemia are major risk factors for vascular calcification and coronary artery disease, the major cause of death in patients with chronic kidney failure [2-4]. Therefore, a vitamin D analog that suppresses the secondary hyperparathyroidism with low calcemic and phosphatemic activity would be of interest.

The two vitamin D analogs available in Denmark are alfacalcidol (1α(OH)D_3_) and paricalcitol (19-norD_2_). Their efficacy in suppressing hyperparathyroidism without causing hyperphosphatemia and hypercalcemia has never been compared.

A randomised, controlled study comparing paricalcitol and calcitriol found that paricalcitol could control hyperparathyroidism with fewer sustained episodes of hypercalcemia and increased calcium × phosphate product than calcitriol (1,25(OH)_2_D_3_) [5,6]. The lower calcemic and phosphatemic activities of paricalcitol can be attributed to decreased stimulation of bone resorption, and intestinal calcium and phosphate absorption [7,8].

Alfacalcidol is converted to calcitriol by the liver enzyme 25 hydroxylase [9]. If alfacalcidol only has effects when converted into calcitriol, we could apply the data from the existing studies, and expect alfacalcidol to be more calcemic and phosphatemic than paricalcitol.

However, a few studies intimate that alfacalcidol does not need to be hydroxylated into calcitriol before exerting its effects. Alfacalcidol apparently has its own intrinsic effects. With similar doses of alfacalcidol and calcitriol mediating the same degree of suppression of PTH, the peak concentration of 1,25(OH)_2_D_3 _is markedly lower after alfacalcidol administration than after calcitriol administration [10]. Also the suppression of PTH secretion from bovine thyroid glands in vivo, where the renal 25-hydroxylation of alfacalcidol is unavailable, is equal between alfacalcidol and paricalcitol [11].

Previous studies are not able to guide the clinician in choosing between alfacalcidol and paricalcitol when treating the patient with kidney disease and secondary hyperparathyroidism. Furthermore, paricalcitol is more expensive than alfacalcidol and needs greater justification if prescribed.

This is the first clinical study investigating whether there is a difference between the ability of alfacalcidol and paricalcitol to suppress secondary hyperparathyroidism in patients receiving chronic haemodialysis therapy without causing hyperphosphatemia and hypercalcemia. This study has been requested several times both in the literature and in the everyday clinical practice [12]. To address this problem, we are currently recruiting for this non-commercial investigator-initiated study.

## Methods/design

### Participants

Eligible subjects are > 18 years old and receiving chronic haemodialysis therapy for at least 3 month prior to screening in any of nine Danish haemodialysis outpatient clinics. After a period of minimum 6 weeks wash out, without any kind of vitamin D supplement, and sufficiently regulated p-phosphate (<1.8 mmol/l) and ionised p-calcium (<1.25 mmol/l), the patients are candidates for inclusion if p-iPTH is >350 pg/ml (37,1 pmol/l). The maximal allowed dose of calcium-containing phosphate-binders is 1600 mg elementary calcium a day at the time of inclusion. The patients may not be scheduled for parathyroidectomy or planned to start treatment with calcimimetics according to the local guidelines in the participating departments. Anticonception and negative pregnancy test should be present for fertile women. They are not admitted to the study if any of the following criteria are present (1) malignancy during the past five years (2) expected survival less than one year (3) pregnancy or breast feeding (4) allergy to any ingredient of Etalpha^® ^or Zemplar^® ^(5) in treatment with calcimimetics (6) participating in any other clinical trials which could affect the endpoints.

### Interventions

**Figure 1:**
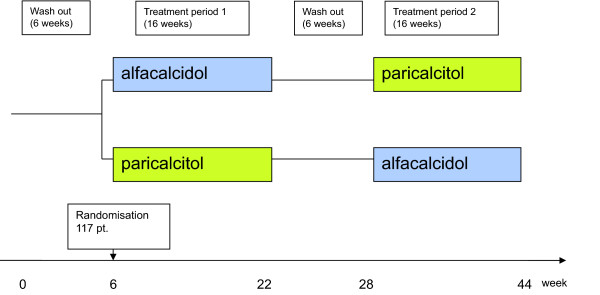
**Flow chart for comparative cross-over study of alfacalcidol and paricalcitol**. After a 6 week wash out period, 117 patients are included. Every participating patient receives 16 weeks of treatment with alfacalcidol and 16 weeks of treatment with paricalcitol. The order of the treatments is decided by randomisation.

The study design is a crossover design where the patients are randomised into two treatment arms (Figure 1). The randomisation is performed after 6 weeks wash out during which the patients are not allowed to receive any kind of vitamin D supplement. The first arm receives alfacalcidol for 16 weeks and then goes through another 6 weeks wash out period before they receive paricalcitol for 16 weeks. The second arm receive paricalcitol for 16 weeks and then goes through another 6 weeks wash out period before they receive alfacalcidol for 16 weeks. The drugs are given at the end of haemodialysis treatment two or three times a week depending on the frequency of haemodialysis treatment. The start dose of alfacalcidol (Etalpha^®^Injection) is 3 μg a week and the start dose of paricalcitol (Zemplar^®^Injection) is 9 μg a week. Every second week the dose is titrated 50% according to p-phosphate, p-calcium and p-iPTH. As long as p-phosphate <1.8 mmol/l and ionised p-calcium <1.30 mmol/l and p-iPTH>150 pg/ml the dose is increased. When p- iPTH ≤ 150 pg/ml and p-phosphate <1.8 mmol/l and ionised p-calcium <1.35 mmol/l the dose is retained. If at any time p-phosphate >1.8 mmol/l or ionised p-calcium >1.35 mmol/l in two repeated measurements the dose is reduced.

After inclusion the dose of calcium-containing phosphate-binders can only be unchanged or reduced. Elevated p-phosphate should be treated thoroughly with calcium-free phosphate-binders, dietary intervention and re-evaluation of the dialysis dose. Elevated p-calcium should lead to dietary intervention and reduction of calcium containing phosphate binders. The calcium concentration of dialysate is fixed to 1.25 mmol/l.

### Objectives

In the current study we test the hypothesis, that there is a difference between the ability of alfacalcidol and paricalcitol to suppress secondary hyperparathyroidism in patients receiving chronic haemodialysis without causing hyperphosphatemia and hypercalcemia.

### Outcomes

The primary efficacy end point is the proportion of patients achieving ≥ 30% reduction in iPTH from baseline until the last 4 weeks of treatment with alfacalcidol or paricalcitol. The secondary outcomes is changes in ionised p-calcium, p-phosphate, calcium × phosphate product, p-alkaline phosphatase, p-25(OH)vitamin D, p-1,25(OH)_2_vitamin D, blood pressure, pulse and pulse pressure from baseline until the end of treatment with alfacalcidol or paricalcitol respectively. The frequency of parathyroidectomy or need for calcimimetics is registered.

Safety endpoints are b-haemoglobin, b-leukocytes, b-trombocytes, p-sodium, p-potassium, p-magnesium, p-bicarbonate, p-creatinine, p-urea, urea reduction rate, p-albumin, p-alanine aminotransferase, p-bilirubin, p-C-reactive protein and weight measured at the beginning and after each treatment period.

All laboratory samples are drawn from the blood lines of the dialyzer before start of the dialysis. P-iPTH, ionised p-calcium and p-phosphate are measured every second week during the treatment periods, the others parameters are measured at the beginning and the end of each treatment period. The urea reduction rate is calculated from a urea measured before dialysis and a urea measured immediately after dialysis. All laboratory analyses are performed at the participating departments' local laboratories.

Blood pressure and pulse are measured after 5 minutes rest before dialysis, and weight is measured before the dialysis session with clothes but no shoes.

### Sample size

We assume that the proportion of patients achieving ≥ 30% reduction in iPTH in the last four weeks of the treatment period would be 50% in the alfacalcidol group and 68% in the paricalcitol group [13,14]. Based on 0.8 power to detect a significant difference (P = 0.05, McNemars test), 117 patients is required for inclusion.

### Randomisation

A randomisation list is computer generated by an external statistician. The patients are randomised in blocks of ten, to secure equal distribution in the two groups in each department. Assignments are enclosed in sequentially numbered, opaque, sealed envelopes. When a patient is included an envelope is opened sequentially and the patient's initials and identification number written on the assignment. The patients are enrolled and treatment assignment ascertained by the primary investigator or any of his/her delegated at each centre. The envelopes are generated by the coordinator and afterwards the allocation list is packed in a sealed, opaque envelope and stored by the coordinator.

### Blinding

This is an open-label study.

### Statistical methods

The frequency of reaching a 30% reduction in iPTH for a minimum of 4 weeks is compared between the two treatments in the same person by McNemar test for paired observations. It is tested whether the effect is dependent of the order of the periods.

Furthermore the effect of the two treatments after the first period will be analysed separately from the second period.

The distribution of the variables and the changes in the variables from the start until the end will be described. Mean, median, standard deviation and range will be reported.

All tests are two-sided tests α = 0.05. The changes during the two treatments will be compared using tests for paired observations.

Parametric tests (paired t-test) will be used if the distributions are normal and non-parametric test (McNemar test and Wilcoxon signed ranks test) for ordered or continuous data.

All participants that are evaluable will be included in the statistical analysis.

The analysis will be performed in Statistica with the procedures: UNIVARIATA, FREQ and ANOVA.

The data analysis will be performed blinded. While doing the statistical analysis the treatment groups will be unknown.

All data will be described and the reason for missing values will be described. Every violation from the statistical plan will be described in the final publication.

All eligible subjects receives a written description of the study objective, schedule, benefits and risks, other treatment possibilities, funding and contact information.

They are offered a consultation providing further information. Here they can bring their relatives.

If the subject agrees to participate a written informed consent is signed.

The study is in compliance with the Helsinki Declaration and is approved by the Danish National Committee on Biomedical Research Ethics (SJ-27), the Danish Medicines Agency (EudraCT: 2006-005981-37) and Danish Data Protection Agency (2007-41-0503)

## Discussion

This article describes the protocol of a randomised trial comparing the ability of alfacalcidol and paricalcitol to suppress secondary hyperparathyroidism without causing hypercalcemia and hyperphosphatemia in patients with chronic kidney disease. As far as we know, alfacalcidol and paricalcitol have never been compared in a randomised clinical trial before.

The advantage of the cross-over design is an elimination of the stochastic variation due to differences between individuals. This reduces the sample size needed. In Denmark in year 2007, there was 2037 haemodialysis patients and a death rate of 21.7% per year in this patient group [15]. Therefore, limiting the sample size is necessary.

When deciding the length of the treatment period it was a balance between different considerations. First, it should not be too long to avoid too many patients to drop out before completing the study. With a mortality rate of 21.7% a year, this is a problem [15]. Second, the treatment period should be long enough to reach a steady state in parathyroid hormone suppression. Going through other long term interventional studies with alfacalcidol or paricalcitol, 16 weeks seems to be the minimal feasible length [6,16]. Third, there is a risk of progression of the secondary hyperparathyroidism over time. However, secondary hyperparathyroidism is a chronic disease. The disease should not progress in a period of 44 weeks, as long as the patients are treated with some kind of active vitamin D.

The wash out period should be long enough to avoid carry over effect from one period to the other. However, a long period with out vitamin D supplementation would be ethically irresponsible, because of the important bone preserving effects and the possible prophylactic effects against cardiovascular disease. For clinical practice, it is not possible to analyse plasma levels of either paricalcitol or alfacalcidol. The pharmacokinetic data available are therefore based on the decline in plasma 1,25(OH)_2_D_3 _following intravenous injection of the vitamin D analog. The half-life of 1,25(OH)_2_D_3 _after intravenous administration of alfacalcidol is 36 hours and paricalcitol 13-30 hours [10,17]. However, the suppression of parathyroid hormone is much longer lasting with a maximal suppression 24 hours after intravenous alfacalcidol [18], although not described for paricalcitol. Unfortunately, there are not any studies describing the time until normalised PTH. A wash out period 42 times the half-life of the compounds and until maximal effect of the compounds, must be sufficient.

A wash out period of 2-8 weeks is used in earlier studies comparing vitamin D analogs with vitamin D analogs or placebo [6,14,19]. The two therapies given during the first treatment period will be compared, in order to analyze the effect without any influence of an, if present, inadequate wash out period,

The statistical analysis will be performed blinded. The participating patients and study personal are not blinded to the assignment, although it would be preferable. This is a non-commercial investigator-initiated study and unfortunately we did not have the resources to do so. However, the endpoints are laboratory measurements, which are objective parameters and therefore not prone to bias.

The randomisation list is kept by the study coordinator. In a randomised study it would be ideal if an external person had stored it. In this cross-over study where every patient is receiving both drugs and only the order in which the treatments are received is randomised, there is not the same incentive to influence the randomisation for each patient as there is in non cross-over studies. Furthermore, the study coordinator is only involved in the randomisation in one of the participating centres.

The blood samples are analysed continually because of the current dose titration. The analyses are performed at each centre's local laboratory. As this is a cross-over study it should not be a problem with different places analysing the blood samples, since the patient is its own control and we secure that each centre keeps the same analysis method during the whole study.

Secondary hyperparathyroidism is suppressed by vitamin D analogs in patients with chronic kidney disease, and thereby is bone health preserved. Sufficient suppression of hyperparathyroidism avoids the need of parathyroidectomy (surgical removal of the parathyroid glands) in this frail population. The main adverse effects are increasing ionised p-calcium and increasing p-phosphate with increasing doses of vitamin D analog. This is problematic, because high ionised p-calcium and p-phosphate are associated with increased risk of cardiovascular disease [20]. This study compares alfacalcidol and paricalcitol to determine if there is any difference in their ability to suppress the secondary hyperparathyroidism in doses not raising ionised p-calcium and p-phosphate. The results of this study will be important to the every-day clinical practice when treating secondary hyperparathyroidism in patients with chronic kidney disease.

## Abbreviations

iPTH: intact Parathyroid Hormone; PTH: Parathyroid Hormone.

## Competing interests

This study is supported by a research grant of 34.000 US$ from Abbott Laboratories A/S without restrictions on publications. Abbott Laboratories A/S has financed investigator meetings and conference participation at American Society of Nephrology, Renal Week 2008 for DH.

LB has received honorary for lectures from LEO Pharmaceutical, Genzyme, Abbott Laboratories A/S, Swedish Orphan, Amgen and Fresenius. LB has received an educational grant from LEO Pharmaceuticals paying one year salary while writing her thesis. LB has participated in advisory boards for Fresenius and Abbott Laboratories A/S. KR has no competing interests.

## Authors' contributions

KR, DH and LB made the conception and study design. DH drafted the protocol and the protocol was revised critically by KR and LB. DH is primary investigator. DH, KR, LB has taking part in initiating the study and managing the trial.

DH drafted the manuscript and all authors revised it critically for important intellectual contents. All authors amended and approved the final manuscript.

## Pre-publication history

The pre-publication history for this paper can be accessed here:

http://www.biomedcentral.com/1471-2369/10/28/prepub

## References

[B1] Slatopolsky E Brown A Dusso A Pathogenesis of secondary hyperparathyroidism Kidney Int Suppl 1999 73 S14 S19 10.1046/j.1523-1755.1999.07304.x 10633458

[B2] Block GA Hulbert-Shearon TE Levin NW Port FK Association of serum phosphorus and calcium x phosphate product with mortality risk in chronic hemodialysis patients: a national study Am J Kidney Dis 1998 31 607 617 10.1053/ajkd.1998.v31.pm9531176 9531176

[B3] Block GA Klassen PS Lazarus JM Ofsthun N Lowrie EG Chertow GM Mineral metabolism, mortality, and morbidity in maintenance hemodialysis J Am Soc Nephrol 2004 15 2208 2218 10.1097/01.ASN.0000133041.27682.A2 15284307

[B4] Ganesh SK Stack AG Levin NW Hulbert-Shearon T Port FK Association of elevated serum PO(4), Ca x PO(4) product, and parathyroid hormone with cardiac mortality risk in chronic hemodialysis patients J Am Soc Nephrol 2001 12 2131 2138 11562412 10.1681/ASN.V12102131

[B5] Sprague SM Lerma E McCormmick D Abraham M Batlle D Suppression of parathyroid hormone secretion in hemodialysis patients: comparison of paricalcitol with calcitriol Am J Kidney Dis 2001 38 S51 S56 10.1053/ajkd.2001.28110 11689388

[B6] Sprague SM Llach F Amdahl M Taccetta C Batlle D Paricalcitol versus calcitriol in the treatment of secondary hyperparathyroidism Kidney Int 2003 63 1483 1490 10.1046/j.1523-1755.2003.00878.x 12631365

[B7] Brown AJ Finch J Slatopolsky E Differential effects of 19-nor-1,25-dihydroxyvitamin D(2) and 1,25-dihydroxyvitamin D(3) on intestinal calcium and phosphate transport J Lab Clin Med 2002 139 279 284 10.1067/mlc.2002.122819 12032488

[B8] Finch JL Brown AJ Slatopolsky E Differential effects of 1,25-dihydroxy-vitamin D3 and 19-nor-1,25-dihydroxy-vitamin D2 on calcium and phosphorus resorption in bone J Am Soc Nephrol 1999 10 980 985 10232683 10.1681/ASN.V105980

[B9] Holick MF Semmler EJ Schnoes HK DeLuca HF 1 -Hydroxy derivative of vitamin D 3: a highly potent analog of 1,25-dihydroxyvitamin D 3 Science 1973 180 190 191 10.1126/science.180.4082.190 4348463

[B10] Brandi L Egfjord M Olgaard K Pharmacokinetics of 1,25(OH)(2)D(3) and 1alpha(OH)D(3) in normal and uraemic men Nephrol Dial Transplant 2002 17 829 842 10.1093/ndt/17.5.829 11981071

[B11] Nielsen PK A direct inhibitory effect of 1α-hydroxyvitamin D_3 _on PTH secretion from bovine parathyroid glands J Am Soc Nephrol 1997 8 578A

[B12] K/DOQI clinical practice guidelines for bone metabolism and disease in chronic kidney disease Am J Kidney Dis 2003 42 S1 201 14520607

[B13] Brandi L Daugaard H Nielsen PK Jensen LT Egsmose C Olgaard K Long-term effects of intravenous 1 alpha (OH)D3 combined with CaCO3 and low-calcium dialysis on secondary hyperparathyroidism and biochemical bone markers in patients on chronic hemodialysis Nephron 1996 74 89 103 10.1159/000189286 8883025

[B14] Martin KJ Gonzalez EA Gellens M Hamm LL Abboud H Lindberg J 19-Nor-1-alpha-25-dihydroxyvitamin D2 (Paricalcitol) safely and effectively reduces the levels of intact parathyroid hormone in patients on hemodialysis J Am Soc Nephrol 1998 9 1427 1432 9697664 10.1681/ASN.V981427

[B15] The Danish Society of Nephrology Danish National Registry Annual Report 2007 http://www.nephrology.dk 10.1159/000168465

[B16] Brandi L Daugaard H Tvedegaard E Nielsen PK Egsmose C Storm T Long-term suppression of secondary hyperparathyroidism by intravenous 1 alpha-hydroxyvitamin D3 in patients on chronic hemodialysis Am J Nephrol 1992 12 311 318 10.1046/j.1525-139X.2002.00086.x 1488999

[B17] Bailie GR Johnson CA Comparative review of the pharmacokinetics of vitamin D analogues Semin Dial 2002 15 352 357 10.1159/000083979 12358640

[B18] Brandi L Egfjord M Olgaard K Comparison between 1alpha(OH)D3 and 1,25(OH)2D3 on the suppression of plasma PTH levels in uremic patients, evaluated by the 'whole' and 'intact' PTH assays Nephron Clin Pract 2005 99 c128 c137 10.1053/ajkd.1998.v32.pm9808143 15722644

[B19] Llach F Keshav G Goldblat MV Lindberg JS Sadler R Delmez J Suppression of parathyroid hormone secretion in hemodialysis patients by a novel vitamin D analogue: 19-nor-1,25-dihydroxyvitamin D2 Am J Kidney Dis 1998 32 S48 S54 10.1097/01.ASN.0000133041.27682.A2 9808143

[B20] Block GA Klassen PS Lazarus JM Ofsthun N Lowrie EG Chertow GM Mineral metabolism, mortality, and morbidity in maintenance hemodialysis J Am Soc Nephrol 2004 15 2208 2218 15284307 10.1097/01.ASN.0000133041.27682.A2

